# Evaluation of an Antioxidative Thermoresponsive Polydiolcitrate Hydrogel in a Novel Diabetic Pig Impaired Wound Healing Model

**DOI:** 10.1007/s40883-025-00425-w

**Published:** 2025-06-25

**Authors:** Maria Mendez-Santos, Yunxiao Zhu, Mouhamad Alloosh, Chongwen Duan, Marilene van den Berg, Michael Sturek, Guillermo A. Ameer

**Affiliations:** 1 Department of Biomedical Engineering, Northwestern University, Evanston, IL United States of America; 2 CorVus Biomedical, LLC, Crawfordsville, IN USA; 3 CorVus Foundation, Inc., Crawfordsville, IN USA; 4 Department of Surgery, Feinberg School of Medicine, Chicago, IL USA; 5 Center for Advanced Regenerative Engineering, Northwestern University, Evanston, IL USA; 6 Querrey Simpson Institute for Regenerative Engineering at Northwestern University, Northwestern University, Chicago, IL USA

**Keywords:** Ossabaw miniature swine, Delayed wound healing, Pig model, Chronic wounds, Citric acid

## Abstract

**Purpose:**

Non-healing chronic wounds in diabetic patients pose a significant health and economic burden. We have previously shown that the citrate-based thermoresponsive macromolecule poly(polyethylene glycol citrate-co–N-isopropylacrylamide) displaying the laminin-derived peptide A5G81 (A5G81-PPCN) accelerates wound closure when used as a regenerative dressing in diabetic mice. Although results are promising, A5G81-PPCN should be evaluated in a relevant large animal model of impaired wound healing. While several large animal models of impaired wound healing have been reported, the Ossabaw miniature swine is unique because it exhibits the full spectrum of metabolic syndrome features and vascular complications that are most similar to those of humans. In this study we investigated whether alloxan-induced diabetic Ossabaw miniature swine would manifest impaired wound healing similar to that observed in humans and evaluated the efficacy and safety of A5G81-PPCN, PPCN, and the commercial dressing Promogran Prisma™.

**Methods:**

After at least 5 months of hyperglycemia (≥ 200 mg/dL) eight full-thickness wounds (3 cm × 3 cm × 5 mm) were created on the back of each animal. Weekly dressing changes, treatment reapplications, and monitoring of blood glucose and weight were performed for 8 weeks post-wounding.

**Results:**

Diabetic Ossabaw swine exhibited notable delayed healing compared to non-diabetic counterparts, validating the model’s relevance. Moreover, PPCN and A5G81-PPCN exhibited accelerated wound closure rates relative to Promogran Prisma™.

**Conclusion:**

This research underscores the potential for this citrate-based thermoresponsive macromolecule to address an unmet clinical need for healing wounds in diabetic patients and highlights Ossabaw swine as a new model for studying impaired wound healing in diabetes.

**Lay Summary:**

Non-healing chronic wounds in diabetic patients pose a significant health and economic burden. We previously developed a unique regenerative dressing called A5G81-PPCN, made from a temperature-sensitive material with a peptide component that accelerates wound healing in diabetic mice. To test this dressing in a clinically relevant model, we used Ossabaw miniature pigs, which mimic human metabolic syndrome and blood vessel complications. After inducing diabetes in these pigs, we created skin wounds and monitored healing for eight weeks. Results show that A5G81-PPCN and PPCN dressings accelerate wound closure relative to a commercial dressing, Promogran Prisma™. This research suggests that A5G81-PPCN could be a valuable new approach to help heal diabetic wounds and positions the Ossabaw pig as an important model for studying diabetic wound healing.

**Supplementary Information:**

The online version contains supplementary material available at 10.1007/s40883-025-00425-w.

## Introduction

Impaired wound healing is a worldwide problem, characterized by the inability of the wound to heal in a timely manner. It is estimated that 1–2% of the world’s population suffer from chronic wounds [1]. Moreover, this problem affected 10.5 million people in US population in 2019, 3.8 million people in the UK population in 2018, and approximately 30 million people in China in 2020 [2–5]. The economic impact is significant with Medicare patients accounting for approximately $22.5 billion in 2019, National Health Service (NHS) for £8.3 billion (11.122 billion USD) in 2018, and 55,270 RMB Yuan (8,010 USD) in 2018 [2, 3, 6]. These wounds result from complications associated with different chronic conditions, such as obesity, peripheral artery disease, heart disease, and diabetes [7]. Diabetes is the leading cause of lower-limb amputation, or the surgery to remove a toe, foot, or leg, in the United States, with complications from diabetes encompassing 80% of all lower-limb amputations. The incidence and prevalence of diabetes are increasing with an estimated 37.3 million Americans (about 1 in 10 people) having diabetes and about 38% of the adult population having prediabetes [8]. Furthermore, according to the World Health Organization (WHO), diabetes was directly responsible for 1.5 million deaths, with 48% of these fatalities occurring before the age of 70 [9]. Diabetes causes blood vessels to narrow, reducing blood flow and impairing the wound healing process. These complications include reduced release of chemokines and growth factors, impaired angiogenesis, prolonged inflammation, and increased oxidative stress [10, 11]. The heightened oxidative stress, along with impaired angiogenesis and exacerbated inflammation in the wound bed, further disrupts healing [12]. Studies have shown that diabetic fibroblasts exhibit reduced proliferation, migration, and responsiveness to cytokines and growth factors such as TGF-β and PDGF [13, 14]. In contrast, diabetic keratinocytes are hyperproliferative but display impaired differentiation and migration. This is particularly concerning because diabetes weakens the immune system, and when coupled with chronic inflammation, it significantly increases the risk of infection.

We have previously demonstrated that the citrate-based thermoresponsive macromolecule, poly(polyethylene glycol citrate-co–N-isopropylacrylamide) (PPCN), functionalized with the laminin-derived peptide A5G81 (A5G81-PPCN), accelerates wound closure when used as a regenerative dressing in diabetic mice. PPCN is an antioxidant macromolecule that is thermoresponsive at physiologically relevant temperatures, forming a hydrogel that conforms to the wound site [15]. The citric acid backbone helps mitigate oxidative stress, while the A5G81 peptide provides biological cues to enhance cellular migration and proliferation of human dermal and epidermal cells. Although the results are promising, A5G81-PPCN should be evaluated in a relevant large animal model of impaired wound healing [16].

Several large animal models have been developed to study the impaired healing caused by diabetes mellitus including the use of dogs, monkeys, and pigs [17, 18] that undergo chemical, genetic, and surgical (pancreatectomy) manipulations. Hadley et al. reported that dog skin is generally looser and more elastic, which affects wound healing due to a higher degree of contraction. Studies show that dogs achieve 65–72% of healing through contraction, compared to 25–50% in humans [19, 20]. Dogs have a more pronounced stratum corneum and variations in hair follicle density, which can influence skin barrier function and susceptibility to infections [21]. Moreover, the expression of antimicrobial peptides, such as canine β-defensin 103, plays a key role in skin defense mechanisms, and these may differ in both quantity and efficacy compared to humans, potentially impacting the wound healing process and infection control [22]. Non-human primates (NHPs), while possessing a comparable epidermal structure, differ significantly in overall skin thickness and the presence of specific skin appendages, which can affect the translational relevance of findings [23]. In contrast, pigs possess skin that is structurally and functionally more similar to human skin in terms of histological features, such as epidermal thickness and dermal structure [24]. This similarity is critical for wound healing studies, as it allows for more accurate modeling of human skin responses to various treatments. O’Brien et al. asserted that pigs are widely regarded as the optimal model for wound healing studies due to their comparable healing mechanisms, including scar tissue formation and the inflammatory response [25]. Swine models are most commonly used because they are more cost effective and there are fewer ethical concerns and public sensitivity about their use compared to the use of NHPs [26].

In swine, wounds are typically made in the dorsal side as it has a more substantial epidermal structure compared to the abdomen or groin region. Moreover, wounds in areas that are subject to motion have more dynamic tension can lead to contraction, as variations in mechanical stress can significantly influence wound healing dynamics, including re-epithelialization and wound contraction [24]. The lack of mechanical stress allows for more reliable experimental observations [27, 28]. The dorsal region is also preferable for experimental wounds as it is more accessible, which facilitates controlled wound creation, monitoring, and treatment. Furthermore, using the dorsal area for thick wounds aligns with ethical practices in animal research, as it reduces the discomfort and risk to the animal [28].

Several impaired healing porcine models have been developed such as Yorkshire, Yucatan, Lanyu, Landrace, Göttingen, and Sinclair. The Yorkshire and Yucatan, which are commonly used, exhibit healing times of 12–14 days and 16 days post-wounding, respectively[27, 29–31]. If these animals are induced to be diabetic, they have delayed healing within 18 to 35 days post-wounding. Ossabaw miniature swine can typically heal in 30 days post-wounding. Similarly, in humans, chronic wounds—such as venous ulcers and diabetic foot ulcers- can take months to years to heal, depending on the wound type and location. The likelihood of healing decreases if the wound is present for more than 6 months. A clinical study by Borda et al. found that chronic leg ulcers took approximately 200 days to heal ulcers, with other studies indicating a mean time range of 3–12 months [32]. Although the various swine breeds can exhibit impaired healing, the Ossabaw stands out for its ability to develop more complex metabolic profiles as it has all the hallmarks of metabolic syndrome (MetS).

MetS is defined by several interrelated factors including obesity, dyslipidemia (abnormal lipid levels), hypertension (high blood pressure), insulin resistance, glucose intolerance, and elevated levels of proinflammatory markers in the bloodstream. [33–36]. The Ossabaw swine can develop all the criteria for MetS when subjected to a diet high in calories, fat, fructose, and cholesterol. The Ossabaw swine stands out as the most extensively studied and characterized model of MetS, exhibiting high relevance to human pathology [36]. It is important to note that some of these breeds, such as the Göttingen, can develop MetS without secondary comorbidities such as nonalcoholic steatohepatitis (NASH), heart failure, and other vascular consequences [36]. The Ossabaw pig’s natural susceptibility to metabolic syndrome that resembles MetS in humans make it a superior model compared to other breeds like the Sinclair, Yorkshire, Yucatan, Landrace, and Göttingen [36]. We are studying type 1 diabetic Ossabaws with profound fasting hyperglycemia in this initial study. A future study might include robust MetS conditions in the Ossabaw pig along with profound fasting hyperglycemia.

In this study we investigated whether alloxan-induced diabetic Ossabaw miniature swine would manifest impaired wound healing similar to that observed in humans and evaluated the efficacy and safety of A5G81-PPCN, PPCN, and the commercial dressing Promogran Prisma™. We show that PPCN and A5G81-PPCN can conform to the wound, undergo a rapid and reversible phase transition from liquid to gel at physiologically relevant temperatures, [16] and accelerate wound closure relative to saline and Prisma™.

## Results

**Fig. 1:**
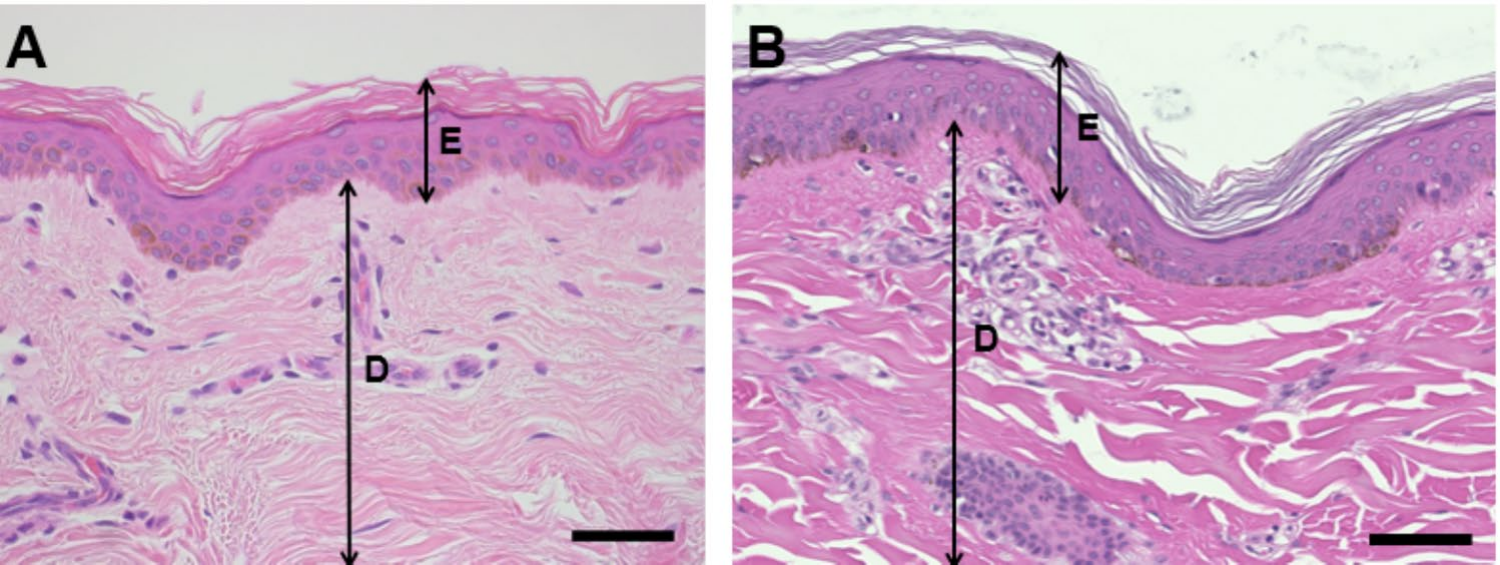
Histological comparison of (**A**) human skin and (**B**) Ossabaw pig skin. The images show the structural similarities between the two species’ skin. The epidermis (**E**) and dermis (**D**) layers are labeled in each sample (scale bar = 50 µm)

**Fig. 2:**
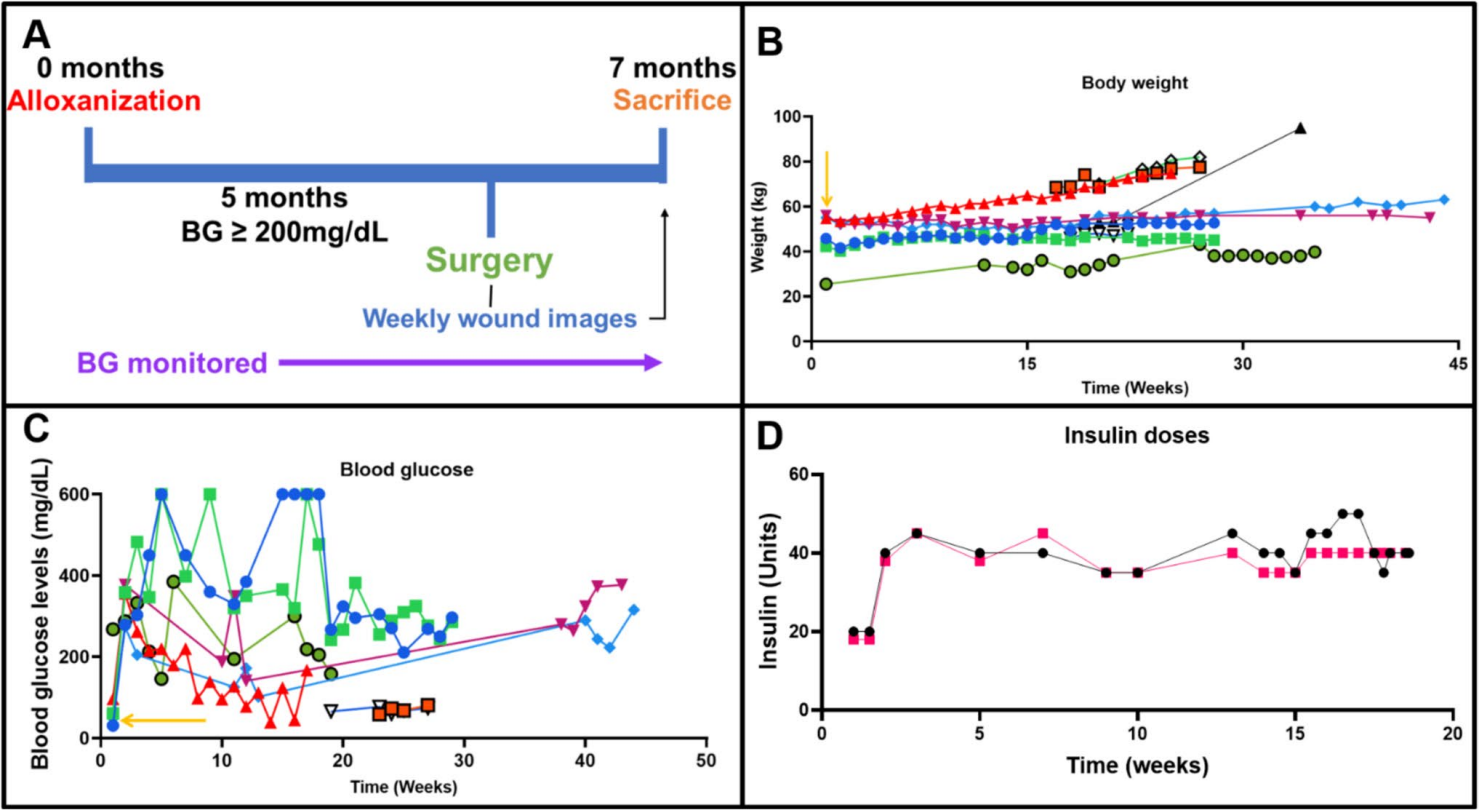
Diabetes Induction Timeline, Body Weight and Blood Glucose Monitoring, and Insulin Administration in Pigs (**A**) Timeline of diabetes induction and study. (**B**) Body weight monitoring for 10 pigs. The yellow arrow indicates Alloxan administration. Diabetic pigs are represented by lines with blue circles, green squares, purple triangles, blue diamonds, and green circles; the mildly diabetic pig by the line with red triangles; and non-diabetic pigs by lines with blue triangles, black triangles, red squares, and green diamonds. (**C**) Blood glucose levels over time. Severely diabetic pigs (blue circles, green squares, purple triangles, blue diamonds, and green circles) and the mildly diabetic pig (red triangles) are compared with non-diabetic pigs (red squares and blue triangles). The yellow arrow marks the time of Alloxan administration. (**D**) Insulin administration schedule for two severely diabetic pigs requiring insulin to maintain body weight (positive energy balance)

*The Ossabaw pig is suitable to study diabetes-impaired wound healing.* Histological examination of human and Ossabaw pig dorsal skin revealed similar epidermal and dermal layer distributions (Fig. 1). To create the diabetic Ossabaw model, the pigs were administered alloxan (125 to 175 mg/kg of body weight) as previously described [33] to induce the destruction of pancreatic beta cells that normally produce insulin. The overall experimental timeline of the study includes both the induction of diabetes and the surgery (Fig. 2A). Wound surgery commenced once the pigs maintained a blood glucose level exceeding 200 mg/dL for at least five months. For the purpose of this study, pigs were classified as diabetic if their blood glucose exceeded 200 mg/dL. One pig that partially recovered from alloxan treatment and was considered “mildly” diabetic. Therefore, its data are included in the supplemental information. The maintenance of body weight verified that pigs were able to maintain a positive energy balance, i.e. not undergo body waste (Fig. 2B). Subsequently, some pigs required insulin administration, and their blood glucose was monitored (Fig. 2C-D). Blood samples were collected to conduct a complete blood count test and ensure that despite elevated blood glucose levels (BG), other markers such as white blood cells (WBC), hemoglobin (HGB), platelets (PLA), blood urea nitrogen (BUN), and creatinine (CRE), remained within typical ranges, as shown in Table 1. Additionally, the white blood cell count from the complete blood count confirms that the animals did not have any signs of systemic infection after surgery.

**Table 1:** Comparative analysis of severely diabetic pigs (*n* = 5) and non-diabetic (*n* = 4) reference values (mean ± standard deviation). Note: Animals were under anesthesia when blood samples were acquired

Parameters	Non-diabetic	Severely Diabetic	*P*-value
Blood glucose	130.5 ± 63.2 mg/dL	411 ± 199.8 mg/dL	0.0320 (*)
White blood cells	8.9 ± 1.4 × 10^3^/µL	14.8 ± 6.84 × 10^3^/µL	ns
Red blood cells	5.6 ± 0.8 × 10^6^/µL	5.9 ± 0.89 × 10^6^/µL	ns
Hemoglobin	11.3 ± 1.1 g/dL	11.3 ± 1.9 g/dL	ns
Platelets	238 ± 85.2 × 10^3^/µL	351.8 ± 187.7 × 10^3^/µL	ns
Blood urea nitrogen	10.5 ± 0.6 mg/dL	15.4 ± 6.3 mg/dL	ns
Creatinine	1.1 ± 0.14 mg/dL	1 ± 0.2 mg/dL	ns

**Fig. 3:**
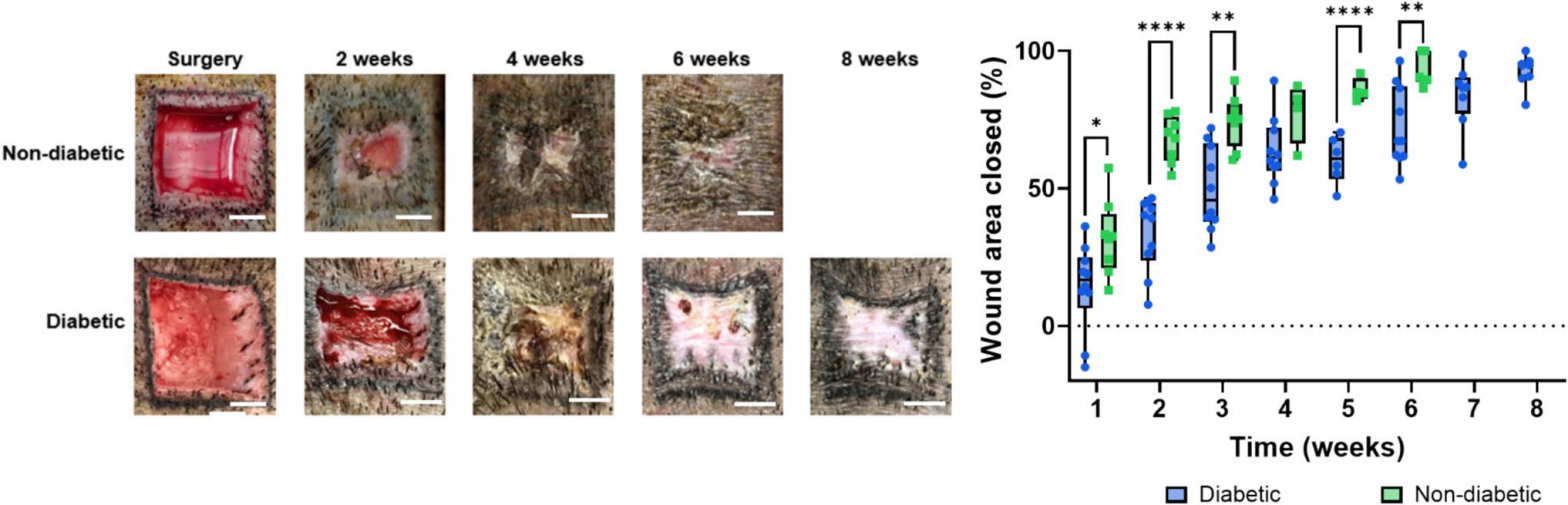
Wound healing over time in non-diabetic and diabetic Ossabaw pigs. (**a**) Representative images of the non-diabetic and diabetic healing process demonstrating impaired healing. (**b**) Quantification of the healing rate for non-diabetic (green squares) and diabetic (blue circles) healing rates (*n* = 8 for non-diabetic pigs, *n *= 10 for diabetic pigs, *p < 0.05, **p < 0.01, and ****p < 0.0001, scale bar = 1 cm)

We compared the healing rates of diabetic and non-diabetic Ossabaw swine by creating wounds on their backs and treating them with saline (Fig. 3). Diabetic pigs exhibited a significantly slower healing rate than non-diabetic pigs. By week 4, non-diabetic pig had reached 80% reepithelization, whereas the diabetic pigs had achieved only 63% reepithelization. Complete reepithelization was delayed in diabetic pigs, occurring mostly in week 8 with an average closure rate of 93%, in contrast to most of the non-diabetic pigs where it occurred at week 6.

**Fig. 4:**
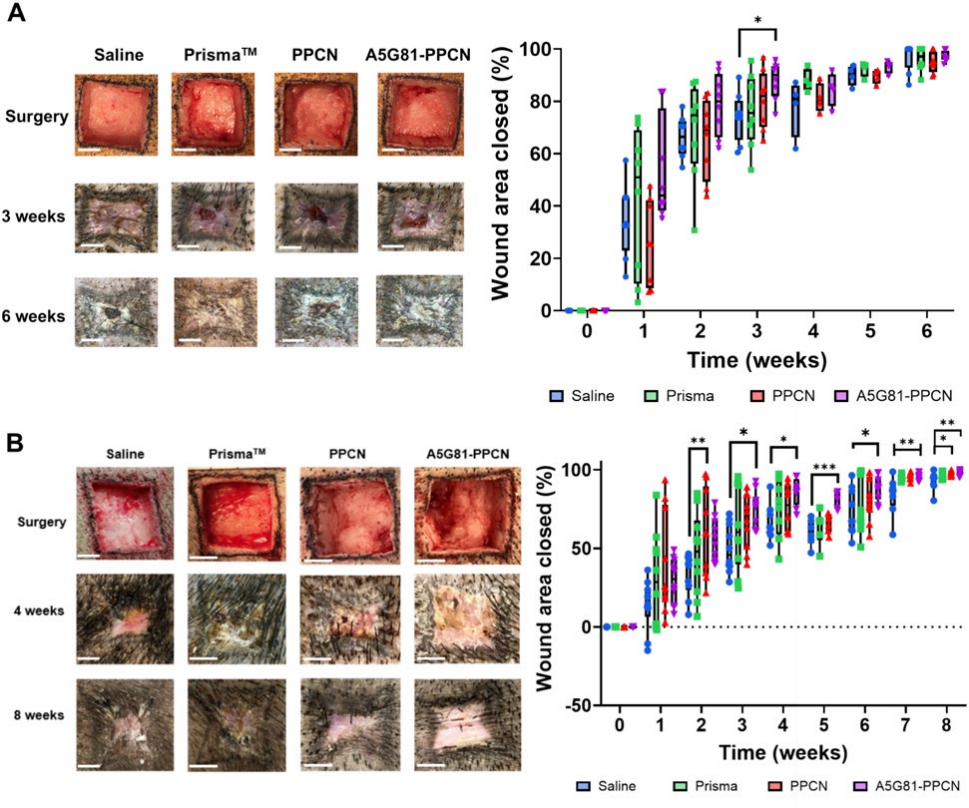
PPCN-A5G81 accelerates wound closure rates. Graphs show the percentage of wound closure over time for each treatment group: (**A**) non-diabetic animals and (**B**) diabetic animals. Treatments were compared to saline. A subzero wound area indicates wound enlargement beyond the initial size. Notably, by week 7, all the treatments are *p* < 0.01 by week 7. (No asterisk = not significant, *p < 0.05, **p < 0.01, ***p < 0.001; scale bar = 1 cm)

*A5G81-PPCN accelerates wound closure in full-thickness wounds in non-diabetic and diabetic pigs.* We had previously observed that A5G81-PPCN could enhance the rate of wound closure, reepithelization, and granulation tissue in diabetic mice [16]. Therefore, we hypothesized that the accelerated wound healing observed in diabetic mice would also be observed in a diabetic large animal model. To test this hypothesis, we investigated wound closure in a full-thickness wound using both non-diabetic (*N* = 4) and severely diabetic (*N* = 5) Ossabaw swine. In this study, all animals received 8 wounds with every pair of wounds being treated with one of the following: A5G81-PPCN, PPCN, Promogran Prisma™ (Prisma™) (3 M Company), or saline. As previously performed, we used Prisma as our positive control as it is one of the commonly used treatments for wounds. In the non-diabetic pigs, the wounds treated with A5G81-PPCN exhibited a remarkable average closure of 87% by week 3, while those treated with PPCN, Prisma, and saline achieved closure rates of 80%, 75%, and 74%, respectively (Fig. 4A). It is worth noting that the contractility of the skin contributed to the accelerated rate of wound closure. Therefore, the use of an impaired healing model would provide insight into the treatment's effectiveness. Subsequently, we conducted the same experiment utilizing the diabetic Ossabaw model. Notably, wounds treated with A5G81-PPCN showed a significantly faster healing process compared to wounds treated with PPCN, Prisma™, and saline. By week 4, these wounds achieved 86% closure, while PPCN, Prisma, and saline treatments reached 76%, 75%, and 63%, respectively (Fig. 4B). But by week 8, A5G81-PPCN and PPCN had achieved complete closure with an average of 98%, whereas Prisma™ and saline had accomplished 96% and 92%, respectively. Noticeably, regardless of the model utilized, A5G81-PPCN accelerated wound closure compared to the other treatments (Fig. 4).

**Fig. 5:**
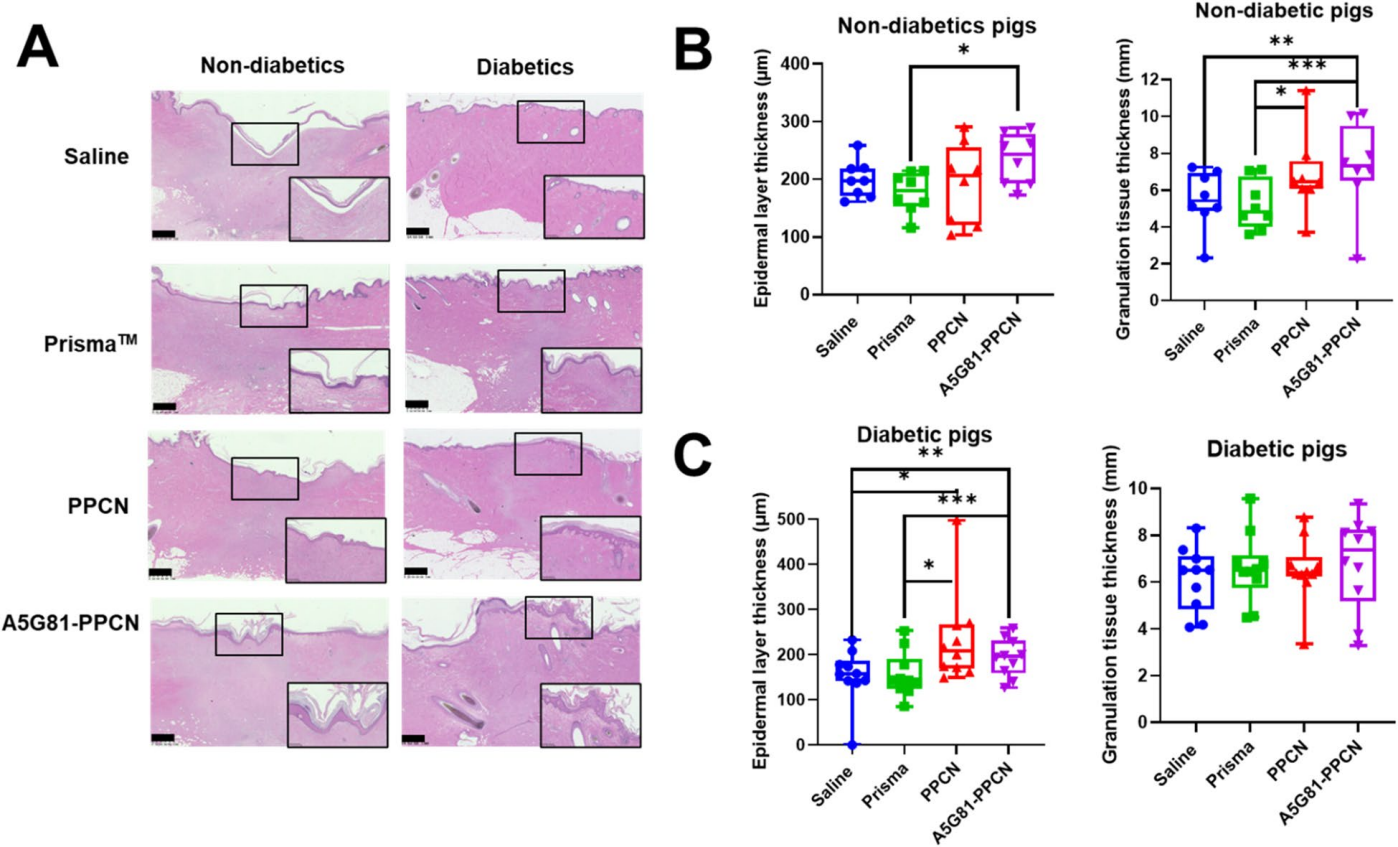
Effects of PPCN and PPCN-A5G81 on granulation tissue thickness and epidermal thickness. **A**) Histological images of tissues samples, stained with hematoxylin and eosin, obtained at week 8 from non-diabetic and severely diabetic pigs. (**B**) Epidermal layer thickness and granulation tissue thickness in non-diabetic pigs (*n* = 8). (**C**) Epidermal layer thickness and granulation tissue thickness in diabetic pigs (*n* = 10; *p < 0.05, ; **p < 0.01, ***p < 0.001; scale bar = 1 mm)

To evaluate tissue maturation, histological assessments of collagen deposition were conducted, including measurements of epithelial thickness (from the stratum corneum to the stratum basale) and dermal thickness (Fig. 5A). For non-diabetic pigs, histological analysis of the healing tissue revealed that A5G81-PPCN treatment resulted in a thicker epidermis when compared to Prisma and thicker granulation tissue when compared to all other groups (*p < 0.05) (Fig. 5B). Epidermal layer thickness was 199.88±58.11µm, 177.38±49.66 µm, 192.92±72.61µm, 214.85±71.86 µm, for saline, Prisma, PPCN, A5G81-PPCN, respectively. Granulation layer thickness was 5.49±1.64 mm, 4.95±2.03 mm, 6.34±2.56 mm, 6.92±2.63 mm for saline, Prisma, PPCN, and A5G81-PPCN, respectively. For diabetic pigs, the epidermal layer thickness was 153.09±69.44µm, 155.86±63.28 µm, 213.84±153.44 µm, 193.46±60.55 µm, for saline, Prisma, PPCN, A5G81-PPCN, respectively. Granulation layer thickness was 6.41±1.52 mm, 6.40±2.00 mm, 6.42±2.00 mm, 7.01±2.01mm, for saline, Prisma, PPCN, A5G81-PPCN, respectively. Both PPCN and A5G81-PPCN significantly enhanced epidermal regeneration compared to saline- and Prisma-treated wounds (*p < 0.05,**p < 0.01) (Fig. 5C)

## Discussion

Diabetes mellitus is a chronic metabolic disorder that poses a significant global health concern due to its rising prevalence. To advance our understanding of diabetes and its underlying pathophysiology, as well as to test novel treatments, it is crucial to employ a clinically relevant large animal diabetic model. In this study, we highlight the utility of the Ossabaw miniature swine as such model. Previously, we demonstrated that wounds treated with A5G81-PPCN exhibit accelerated healing in mice. This study builds on those findings by evaluating the hydrogels in a swine model of diabetes impaired wound healing. Swine have skin that is similar to human skin regarding epidermal thickness, subcutaneous composition, and healing processes. Furthermore, the tight skin resembles that of humans, unlike the looser skin of mice, rats, and rabbits. Our histological findings demonstrate the homogeneity of the skin structures and thickness of the Ossabaw swine skin when compared to human skin as an experimental platform for wound healing, as seen in Fig. 1*.* Moreover, numerous mice or rodents would be required for a comprehensive study; however, far fewer large animals would be used to complete a study to evaluate and compare treatments while minimizing inter-individual variations. In our previous study, we used n ≥ 5 mice, while in this study, only 5 diabetic animals were used. Therefore, this model is crucial for basic and clinical research as it is in accordance with the welfare of animals and the 3Rs of replacement, reduction, and refinement for preclinical trials [28].

While wound healing is a natural process, the rate and extent of regeneration depends on the breed, age, wound size, and location. The thickness of the epidermis and dermis layer, as well as the maturation of the collagen can also differ during different stages of healing. When comparing wound healing rates across different pig models, variations in breed, age, metabolic rate, and overall health status play crucial roles in determining the outcomes. Yorkshire pigs, often favored in research for their larger size and close physiological similarities to human skin, typically exhibit relatively rapid wound healing. Studies by Velander et al. (2008) found that wound closure occurs around 12 to 14 days post-wounding. In contrast, Göttingen minipigs, which are smaller and have a more modest metabolic rate, tend to exhibit slower wound healing, with closure occurring around 21 days post-wounding (Eisler et al., 2024). Other models, such as Yucatan pigs, also demonstrate slower wound healing compared to full-sized breeds. These miniature pigs, known for their docile nature and manageable size, heal at a rate similar to the Göttingen pigs. While they may not heal as quickly as the Yorkshire breed, Yucatan pigs are often used in studies that require long-term observation due to their plateau in growth and ease of handling. Moreover, the Ossabaw pig, another significant model, presents a distinct healing profile. The Ossabaw pig's healing process averages 42 days post-wounding, potentially because of the breed's unique metabolic characteristics, including its thrifty genotype [33].

The metabolic dysregulation associated with diabetes significantly impairs normal wound healing pathways, with the effect being even more pronounced in Ossabaw pigs. Under diabetic conditions, their healing rate extends to 56 days post-wounding, compared to 35 days in Yucatan pigs, as previously mentioned. This extended healing period is crucial as it more accurately reflects the delayed wound healing seen in humans with diabetes and other chronic conditions, where healing can take months to years. As shown in Fig. 3*,* the hyperglycemic state of the animals affects their natural healing rate as the wounds were only treated with saline and resulted in the non-diabetic pigs closing their wounds faster due to in part the ability to contract their skin as elasticity is affected by hyperglycemia. This delay highlights the significant impact that metabolic diseases have on tissue repair and underscores the importance of choosing the appropriate pig model depending on the specific health condition being studied. It is important to note, that Berry et. al. [34] reported that the magnitude of wound contraction in full-thickness wounds is high as fibroblasts are converted into myofibroblasts and collagen undergoes contraction. Moreover, the data presented in Fig. 4 stands as insight into the wound healing process, as we can observe the differences between the treatments. Notably, PPCN-A5G81 and PPCN outperform Prisma™ by closing the wounds the fastest and with least variability, likely due to PPCN’s antioxidant properties.

As previously published, A5G81, a laminin-derived peptide, enhances migration and proliferation of dermal fibroblasts and epidermal cells via its interactions with the integrins α3β1 and α6β1. These integrins have distinct and interconnected roles in cellular adhesion, migration, and signaling related to tissue repair. Integrin α3β1 is needed for cell anchorage and migration at the dermal–epidermal junction and plays a crucial role in epithelial repair processes by facilitating keratinocyte migration. It significantly contributes to wound healing through promoting cellular movements necessary for tissue regeneration [35]. Integrin α6β1 stabilizes the epidermis during wound healing by facilitating keratinocyte adhesion to the basement membrane therefore supporting its integrity throughout the healing processes [36]. Since both integrins are crucial for cell-ECM interactions, their combined function enhances wound healing: α3β1 promotes keratinocyte mobility and migration, while α6β1 provides structural support for tissue stability [35]. When conjugated to PPCN, A5G81 further enhances wound closure, likely due to the synergistic effects of these integrin-mediated mechanisms.

As demonstrated by Faghih and colleagues (2015) [37], an accelerated wound closure rate does not necessarily translate into healthy regenerated skin. To evaluate the impact of the treatments on wound healing, we examined the collagen content and other histological changes in the healing skin using hematoxylin and eosin staining (Fig. 5) and Masson’s trichrome staining (Supplementary Fig.S2 and S3). Consistent with our diabetic mice data, PPCN and A5G81-PPCN enhanced regeneration, as these treatments were associated with a significantly thicker epidermal layer and granulation tissue compared to Prisma™ and saline (Fig. 5B and C). Van Dorp et al. [38–40], all reported faster wound closure and, in most cases, enhanced regeneration in epidermis and granulation tissue utilizing different hydrogels in diabetic [16] models, which aligns with our findings.

There are inconsistencies in other models, as seen in Weiss et al. (2024) [41]—a similar study to that of Abadir et al. (2018) [30], which use the Yucatan diabetic model and similar treatment—which found no significant differences between treatments and control wounds. Although there is a difference in wound size, Abadir and colleagues created 5 cm diameter wounds, while Weiss and colleagues made 2.5 cm wounds, any significant difference should have favored the smaller wounds, as they typically require less time to close due to their size.

Interestingly, as one pig recovered from alloxan administration, we were able to observe the effects of controlled diabetes. This mildly diabetic animal did not experience vascular damage within the modest length of this study and exhibited healing rates similar to those observed in healthy animals (Supplementary Fig. S4). The primary limitation of this study was the number of timepoints; having at least one mid-study timepoint to histologically evaluate wound closure progression would have provided a better understanding of the healing process. Future studies should incorporate wound blood perfusion measurements to better understand the healing process as well as mechanical testing of the regenerated tissue. Furthermore, to refine this model the wound could be induced on the plantar side of the foot, as Fuchs et al. [42] have demonstrated that plantar skin has altered physiology that affects wound healing. Finally, having established the clinical relevance of this model for diabetic wound healing, its resemblance to clinical conditions could potentially be further strengthened by introducing an infection into the wound; as Muntazir Andrabi et al. demonstrated, this is possible by establishing a biofilm for 4 days in the Ossabaw swine [43].

In conclusion, diabetic animal models have played a pivotal role in understanding diabetes, its pathophysiology, and potential treatments. Despite the inherent limitations associated with these models, our findings suggest that the Ossabaw swine can be utilized as a valuable resource for evaluating therapies that address impaired wound healing due to diabetes. In this study, we demonstrate that the use of this animal model successfully replicates that extreme hyperglycemia is a major factor in impaired wound healing. This model follows the 3R principles, which is important for animal welfare and essential for clinical translation. Moreover, it also provides a high throughput platform for screening the effectiveness of treatments and histological changes involved in the wound healing process. Furthermore, our findings indicate that wounds treated with A5G81-PPCN exhibit accelerated wound healing that compares favorably with the rates reported by Zhu et al. [16]. Therefore, our results in both small and large diabetic animal models suggest that a PPCN-based dressings may be suitable for humans and improve healing rates.

## Materials and Methods

To study wound healing, we previously developed a thermoresponsive regenerative dressing A5G81-PPCN^19−20^. The dressing was synthesized as previously described by us; briefly, the antioxidant macromolecule PPCN was synthesized via polycondensation and free-radical reaction and functionalized with N-β-maleimidopropionic acid hydrazide (BMPH) as a linker^19^. The laminin-derived dodecapeptide A5G81 contains a terminal cysteine and was covalently conjugated to BMPH-PPCN via click chemistry. This peptide assists integrin-mediated spreading, as well as dermal and epidermal cell proliferation and migration leading to faster regeneration in diabetic mice models.

### Experimental Animals

Four non-diabetic Ossabaw porcine (female), five severely diabetic porcine (type 1 diabetes) (female and one castrated male), and one mildly diabetic porcine(female) purchased from Corvus Biomedical, LLC (44.4–65.8 kg, 1-year-old) were used in this study and were acclimated in the animal care facility for 7 days prior to surgery. The complete blood count (cbc) values were analyzed for normality using the Shapiro–Wilk test; additionally, a students’ two-way t-test was performed for statistical analysis (α = 0.05).

### Induction of Diabetes

At Corvus Biomedical, LLC pigs were administered alloxan (125 to 175 mg/kg of body weight) to induce the destruction of pancreatic beta cells. The blood glucose was monitored and the pigs were received when consistent hyperglycemia (> 200 mg/dl) was detected. The animals were held in compliance with the standards of the current version of the Animal Welfare Act and all the experimental procedures were approved by the Institutional Animal Care and Use Committee of Northwestern University (protocol IS00004924) and animal work was performed at the Center for Comprehensive Medicine at Northwestern University (Chicago, IL).

### Surgical Procedure

The pigs were fasted 12 h prior to being administered isotonic fluids (LRS, 0.9% Sodium Chloride) and sedated intramuscularly with BAM (Butorphanol, Azaperone, and Medetomidine) (0.05–0.2 mg/Kg) and isoflurane (2–4%) via inhalation. Eight square-shaped outlines were tattooed as permanent references of the initial wound edges. Following this, full-thickness wounds of 3 × 3 cm size, and 3 cm between the wounds were created in the back of each animal, as shown in Fig. 4. These wounds were approximately 5 mm in depth to expose the muscle as the subcutaneous fat was removed. Finally, wounds were covered with Tegaderm™ (3 M Company, St. Paul, MN) and an animal jacket was placed to protect the wound.

### Wound Dressing and Measurements

Blood glucose, weight, change the dressing, and reapply treatments were monitored throughout the study. Wounds were treated and evaluated weekly. During dressing changes, wounds were cleaned with saline and photographed before reapplying treatment. A volume of 2.5 mL of PPCN or A5G81-PPCN were applied to each wound, the animal was tented with a heating blanket from above and gelation occurred within 1 min as the color changed from transparent to white as the material crosslinked. In addition, wounds from the other experimental groups were covered by a piece of PRISMA cut to fit the area or treated with only Saline. All treated wounds were then covered with Tegaderm. Those animals that had glucose higher than 300–350 mg/dL were given 6–10 units of insulin (Lantus, 100units/kg) subcutaneously.

### Wound Healing Assessment

Wound closure is a key parameter of wound healing; therefore, it was monitored over time. There are two common methods to assess wound healing: by measuring wound area or wound volume. While volume measurement accounts for wound depth and is more reflective of healing by secondary intention, it can be difficult to measure and interpret in irregularly shaped wounds. The wound area method has been previously reported in mice using the splint technique by Galiano et al. [44], by our group Zhu et al. [16] and most recently by Andrabi et al. [43]. In this study, we assessed wound area using standard digital image analysis with ImageJ, following the approach described by Kuo et al. [28]. A black-ink tattoo was used to mark the edge of each unclosed wound and digital images of the wound area were taken every week and quantified using ImageJ’s freehand selection tool (Supplementary Fig.S5) (Bethesda, MD, USA). The changes in the wound closure were calculated using the following formula.

$$Wound\;closure\left(\%\right)=\left(\frac{Area\;T_0-{Area\;T}_x}{Area\;T_0}\right)\times100$$

where $Area {T}_{0}$ is the initial wound area and ${Area T}_{x}$ is wound area at time x.

### Histological Studies

Samples were fixed in 10% formaldehyde for 72 h and were embedded in paraffin. The blocks were sectioned and stained for hematoxylin/eosin (H&E) for general observation and Masson's Trichrome to observe collagen. Slides were imaged using a NanoZoomer Digital slide scanner (Hamamatsu Photonics, Shizuoka, Japan). The granulation tissue was quantified by measuring the thickness at 5 evenly spaced locations from the center of the wound for each animal using NDP.view2 (Hamamatsu Photonics, Shizuoka, Japan) by two blinded individuals. Human skin/tissue samples were not used in the experiments, the figure of human skin histology was provided by Northwestern University’s Skin Biology & Diseases Resource-Based Center of the National Institutes of Health. The donor was a 32-year-old African American female, with a registration number of NUTC4813.23. The donor had signed an informed consent which was approved by the Institutional Research/Human Ethics Committee No. from the IRB ID# STU00009443.

### Statistical Methods

GraphPad Prism 10 software was utilized to conduct one-way ANOVA tests to evaluate variations in experiments containing multiple datasets. Subsequently, a Bonferroni test was executed on groups displaying significant distinctions to address the issue of multiple pairwise comparisons. Student t-tests were also used as indicated in each figure for statistical analysis among and between groups, and the significance were expressed as follows; *p < 0.05, **p < 0.01, ***p < 0.001, and ****p < 0.0001.

## Supplementary Information

Below is the link to the electronic supplementary material.

Supplementary file1 (PDF 323 KB)

## Data Availability

All data presented in the figures and Supplementary Figures are available from the corresponding author upon reasonable request.

## Declarations

The animals were held in compliance with the standards of the current version of the Animal Welfare Act and all the experimental procedures were approved by the Institutional Animal Care and Use Committee of Northwestern University (protocol IS00004924) and animal work was performed at the Center for Comprehensive Medicine at Northwestern University (Chicago, IL).
