# Supplementary material for: Evaluation of an Antioxidative Thermoresponsive Polydiolcitrate Hydrogel in a Novel Diabetic Pig Impaired Wound Healing Model
Source: Regen Eng Transl Med. 2025 Jun 25;11(4):1013–23. doi: 10.1007/s40883-025-00425-w (PMC12852258; doi:10.1007/s40883-025-00425-w)
Supplement: Supplementary file 1 — Supplementary file1 (PDF 323 KB) [file 40883_2025_425_MOESM1_ESM.pdf]

Supplementary figures:

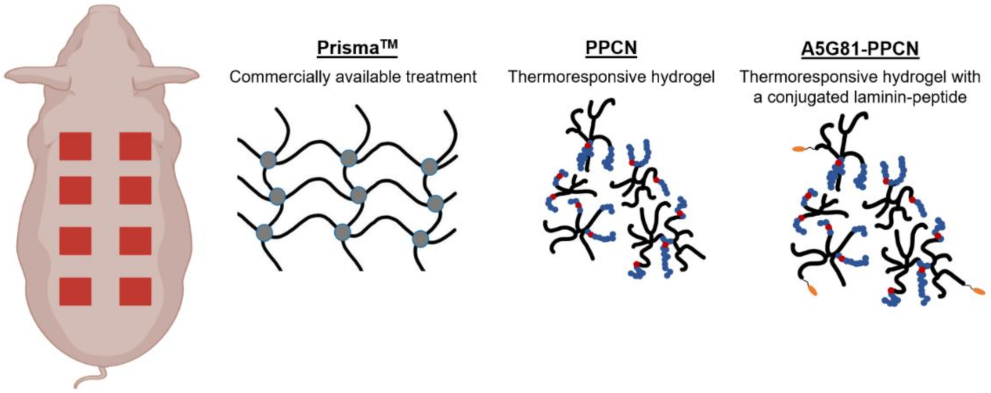

Fig. S1 Schematic representation of the experimental setup. Each animal received 8 wounds, with every pair of wounds treated with one of the following: saline, Prisma^{TM}, PPCN, and A5G81-PPCN. (Pig illustration created with BioRender.com)

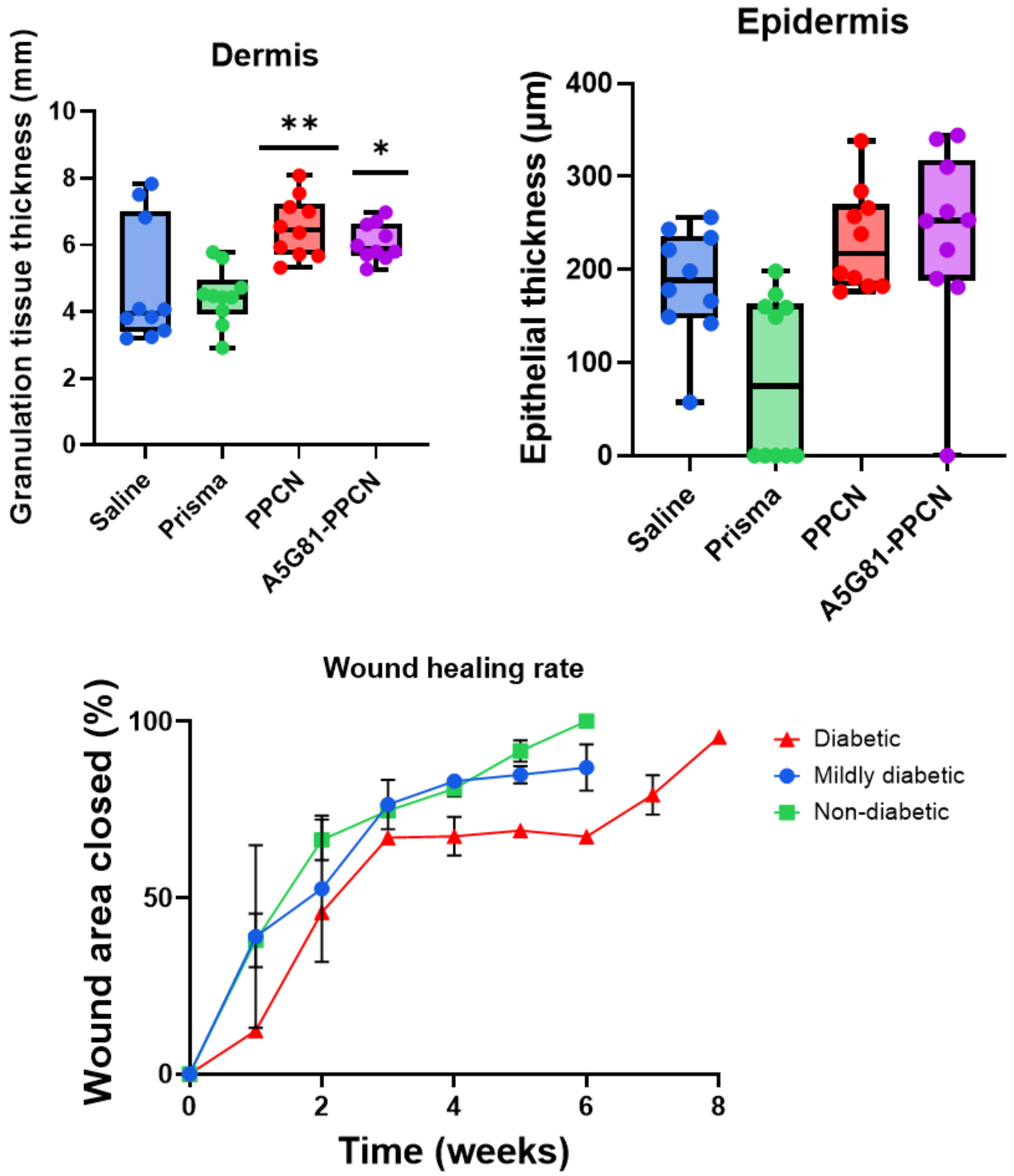

Fig. S2 Mildly diabetic pig. Quantification of histology of wound reveals that wounds treated with PPCN-based therapies exhibit notable increases in epidermal thickness compared to those treated with Prisma™ or saline (*$p<0.05$, **$p<0.01$). No significant differences were observed in granulation tissue thickness across the different treatment groups

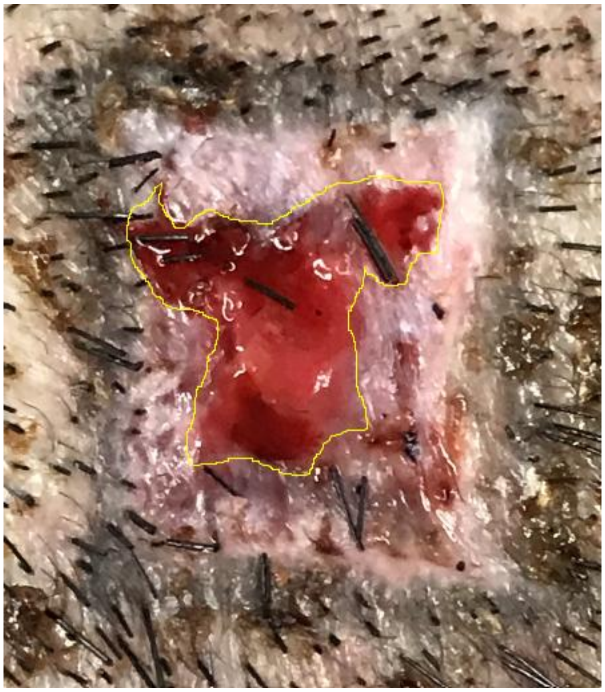

Fig. S3. Example of how wound closure was traced. The open area was outlined using the freehand tool in ImageJ to measure the wounded area.

Pig ID	2721	2711	2876*	2877*
Glucose (mg/dL)	77.0	75.0	178	192
WBC (x10^3/uL)	9.5	10.5	7	8
RBC (x10^6/uL)	5.8	6.6	5	5
HGB (g/dL)	11.9	12.4	10	10
Platelets (x10^3/uL)	310.0	260.0	-	144.0
BUN (mg/dL)	10.0	11.0	11	10
Creatinine (mg/dL)	1.0	1.0	1	1

Table S1 Summary of Complete Blood Count (CBC) Panel for Non-Diabetic Subjects. The table provides an overview of key hematological parameters measured in non-diabetic subjects, including white blood cell count, red blood cell count, hemoglobin, hematocrit, and platelet levels. *Samples were acquired while the animals were under anesthesia. This explains the wide variability in the blood glucose in Table 2.

Pig ID	2698
Glucose (mg/dL)	99
WBC (x10^3/uL)	5.1
RBC (x10^6/uL)	4.0
HGB (g/dL)	7.7
Platelets (x10^3/uL)	17
BUN (mg/dL)	24
Creatinine (mg/dL)	1.3

Table S2 Summary of Complete Blood Count (CBC) Panel for mildly diabetic subject. The table provides an overview of the key hematological parameters measured in pig 2698 that recovered from alloxan and became mildly diabetic as shown in Figure S4., White blood cell count, red blood cell count, hemoglobin, hematocrit, and platelet levels are included.

Pig ID	2660	2667	2826	2872	2958
Glucose (mg/dL)	735.0	461.0	329.0	227	303
WBC (x10^3/uL)	19.7	20.9	7.9	7	19
RBC (x10^6/uL)	5.6	4.8	6.8	7	6
HGB (g/dL)	11.0	9.3	13.2	13	10
Platelets (x10^3/uL)	186.0	446.0	310.0	189.0	628.0
BUN (mg/dL)	25.0	18.0	14.0	10	10
Creatinine (mg/dL)	0.9	0.8	1.0	1	1

Table S3 Summary of Complete Blood Count (CBC) Panel for diabetic subject. The table provides an overview of the key hematological parameters measured in diabetic pigs, including white blood cell count, red blood cell count, hemoglobin, hematocrit, and platelet levels
